# Interaction of the pathogenic mold *Aspergillus fumigatus* with lung epithelial cells

**DOI:** 10.3389/fmicb.2012.00346

**Published:** 2012-09-26

**Authors:** Nir Osherov

**Affiliations:** Department of Clinical Microbiology and Immunology, Aspergillus and Antifungal Research Laboratory, Sackler School of Medicine, Tel-Aviv UniversityRamat-Aviv, Tel-Aviv, Israel

**Keywords:** lung epithelial cells, *Aspergillus fumigatus*, innate immunity

## Abstract

*Aspergillus fumigatus* is an opportunistic environmental mold that can cause severe allergic responses in atopic individuals and poses a life-threatening risk for severely immunocompromised patients. Infection is caused by inhalation of fungal spores (conidia) into the lungs. The initial point of contact between the fungus and the host is a monolayer of lung epithelial cells. Understanding how these cells react to fungal contact is crucial to elucidating the pathobiology of Aspergillus-related disease states. The experimental systems, both *in vitro* and *in vivo*, used to study these interactions, are described. Distinction is made between bronchial and alveolar epithelial cells. The experimental findings suggest that lung epithelial cells are more than just “innocent bystanders” or a purely physical barrier against infection. They can be better described as an active extension of our innate immune system, operating as a surveillance mechanism that can specifically identify fungal spores and activate an offensive response to block infection. This response includes the internalization of adherent conidia and the release of cytokines, antimicrobial peptides, and reactive oxygen species. In the case of allergy, lung epithelial cells can dampen an over-reactive immune response by releasing anti-inflammatory compounds such as kinurenine. This review summarizes our current knowledge regarding the interaction of *A. fumigatus* with lung epithelial cells. A better understanding of the interactions between *A. fumigatus* and lung epithelial cells has therapeutic implications, as stimulation or inhibition of the epithelial response may alter disease outcome.

*Aspergillus fumigatus* is a ubiquitous saprophytic mold with a worldwide distribution (Ben-Ami et al., 2010; McCormick et al., 2010). Inhalation of *A. fumigatus* asexual spores (conidia) can cause a spectrum of clinical manifestations depending on the immunological status of the host (Ben-Ami et al., 2010; McCormick et al., 2010). In the hypersensitive host, conidia inhaled primarily into the bronchial tree can initiate an allergic response culminating in Allergic Bronchopulmonary Aspergillosis (ABPA) (Knutsen and Slavin, 2011). In the immunocompromised host, conidia inhaled mainly into the lung alveoli can cause a life-threatening fungal infection termed Invasive Pulmonary Aspergillosis (IPA) (Hope et al., 2007; Thompson and Patterson, 2011).

In both cases, the initial point of contact between the fungus and the host is a monolayer of lung epithelial cells. Elucidating how these cells react to fungal contact is central to our understanding of Aspergillus-associated diseases. However, remarkably, during the last 20 years, very little research has focused on understanding the interactions between lung epithelial cells and *A. fumigatus*. Less than 40 publications directly dealing with this topic were found during the preparation of this manuscript, compared to many hundreds of papers describing the interactions of *A. fumigatus* with various immune cells.

In contrast, the study of lung epithelial responses to bacterial and viral pathogens is relatively advanced, suggesting that similar progress can be expected in the field of fungal interactions (Evans et al., 2010; Vareille et al., 2011).

In this review, I will outline the main findings to date regarding lung epithelial cell interactions with *A. fumigatus* and identify key areas of research that, in my opinion, need to be further pursued with vigor.

## Bronchial and alveolar epithelium-structure and function

**Figure 1:**
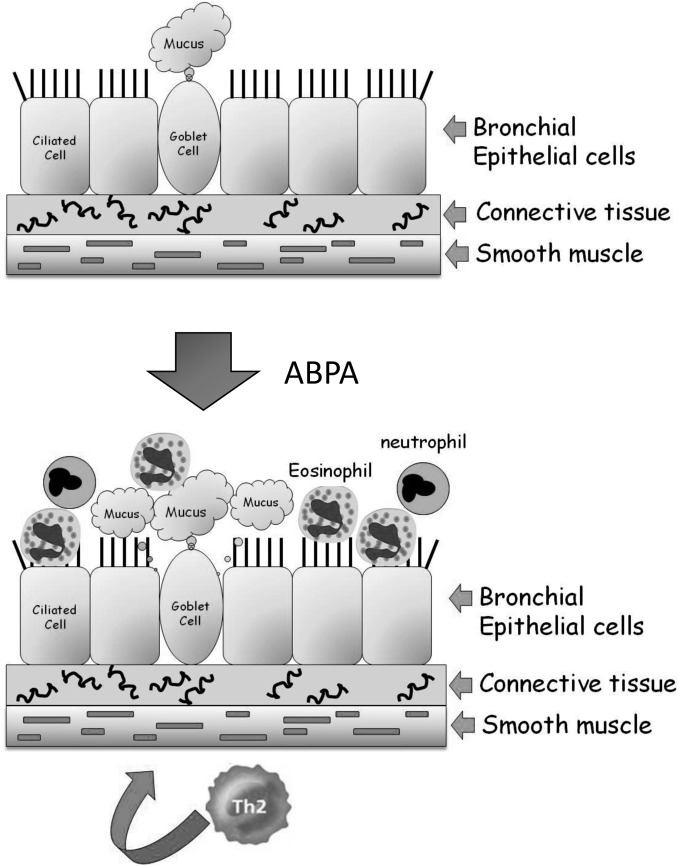
**Structure of the lung airways and the development of ABPA.** The major epithelial cell types in the airways are the ciliated, goblet, and basal cells. They are attached to connective tissue that is surrounded by a band of smooth muscle cells. In ABPA, a Th2-mediated response to inhaled *A. fumigatus* conidia results in enhanced mucus production and the influx of eosinophils and neutrophils from the bloodstream into the airway. These cells inflame and damage the airway, resulting in tissue fibrosis and subsequently bronchiectasis.

The bronchi (singular bronchus) are tubes that allow passage of air into the lung alveoli. They further divide into primary bronchioles and finally into terminal bronchioles connected directly to the alveoli. No exchange of gases takes place in the bronchial structure. A mid-level primary bronchiole is made up of three layers—an inner layer of epithelial cells, a middle layer of connective tissue, and an elastic outer layer of smooth muscle cells (Figure 1). The inner layer of epithelial cells is composed mainly of mucus-secreting goblet cells and ciliated cells that drive the mucus and adherent debris, out of the lungs. In ABPA, inhaled conidia initiate an exaggerated Th-2 mediated inflammatory response. Following activation by T-cell cytokines, epithelial cells release large quantities of proinflammatory cytokines, growth factors and chemokines, amplifying the influx of T-cells, eosinophils, basophils, and other inflammatory cells. This inflammation leads to the associated pathological features of airway hyperresponsiveness, hyperplasia/metaplasia of goblet cells and subepithelial fibrosis (Kato and Schleimer, 2007; Knutsen and Slavin, 2011).

**Figure 2:**
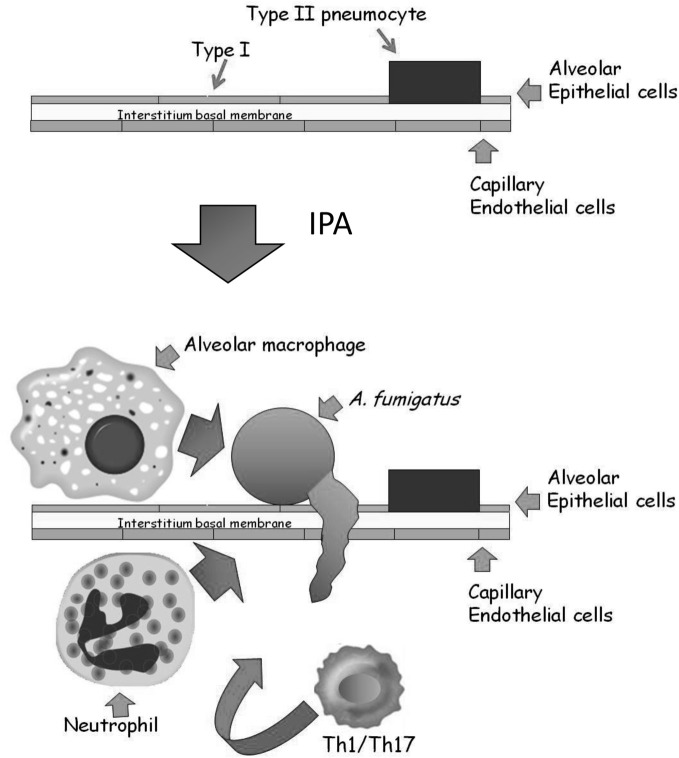
**Structure of the alveolus and the development of IPA.** The major epithelial cell types of the alveolus are the type I and type II pneumocytes. They are attached to a thin basal membrane composed of laminin, collagen, and fibronectin. A single layer of capillary endothelial cells is attached to the lower side of the basal membrane, lining the blood vessel. In IPA, inhaled conidia germinate on the alveolar surface. Immunocompromised patients cannot mount an effective Th1/Th17 based cellular response involving resident alveolar macrophages and infiltrating neutrophils, resulting in conidial germination and hyphal penetration through the thin alveolar wall. Subsequently, profuse hyphal growth blocks the underlying blood vessels, leading to tissue necrosis and ultimately death.

The alveolar epithelium covers a surface area of approximately 100 m^2^ and contains over 700 million individual alveoli, each about 200 microns across. The alveolar membrane is made up of three layers (Herzog et al., 2008) (Figure 2): The inner layer contains two types of epithelial cells: (1) terminally differentiated non-dividing type I cells covering >95% of the inner alveolar surface. These cells are extremely thin (<0.5 microns), enabling rapid gas exchange (2) type II cells, whose primary roles are to secrete surfactant proteins and differentiate into type I cells. Binding of surfactant proteins A and D to *A. fumigatus* conidia enhances phagocytosis and killing by neutrophils and alveolar macrophages (Madan et al., 1997, 2005; Kishor et al., 2002; Pandit et al., 2012). In addition, type II cells secrete cytokines, chemokines, and antimicrobial peptides in response to pathogens (Herzog et al., 2008). The alveolar epithelial cells are attached to a thin basal membrane layer composed of connective tissue proteins including laminin and fibronectin. Attached to the undersurface of the basal membrane is a single layer of capillary endothelial cells, that line the blood vessels coming into contact with the alveoli. In IPA, inhaled conidia, because of their small size (2–3 microns) enter the alveoli and proceed to germinate. The entire alveolar tissue is in most places no wider than 1 micron, so that a fungal germ-tube measuring only 2–3 microns long can easily traverse it and enter the bloodstream. In immunocompetent individuals, resident alveolar macrophages normally ingest and destroy inhaled conidia. If the fungal inoculum is large, they secrete chemokines to recruit circulating neutrophils that can destroy both conidia and growing hyphae. In immunodeficient individuals, these innate immune defenses are lacking or dysfunctional, leading to fungal growth through the alveolar wall into the surrounding blood vessels, causing circulatory obstruction and subsequent tissue necrosis.

## Experimental systems currently used to study the interaction between *A. fumigatus* and lung epithelial cells

The *in vitro* experimental systems developed for studying the interaction between *A. fumigatus* and lung epithelial cells are, for the most part, remarkably basic and simple. Dormant *A. fumigatus* conidia or culture filtrate (CF) secreted by mature mycelium are added to epithelial cells in culture. Most models of bronchial infection use immortalized (i.e., BEAS-2B and HBE) (Balloy et al., 2008; Alekseeva et al., 2009; Fekkar et al., 2012) or cancerous (i.e., H292) (Oguma et al., 2011) bronchial epithelium cell lines. In some cases primary nasal or tracheal cells, usually taken from a biopsy (Amitani et al., 1995; Paris et al., 1997; Botterel et al., 2008) or even whole excised tissue blocks (Amitani and Kawanami, 2009) have been used. Models of alveolar infection have been more limited because of the difficulty in growing primary type I or type II cells in culture. Only very recently has a stable transformed type I cell line been generated but has not yet entered experimental use (Thorley et al., 2011). Due to a lack of a better system, almost all studies of alveolar epithelial infection use the type II-like A549 cell line derived from a lung carcinoma (Lieber et al., 1976), despite the fact that these cells cover less than 5% of the alveolar surface. In addition, A549 cells are hypotriploid and genetically unstable, and when cultured as monolayers, they do not retain the structural or functional characteristic of the original tissue from which they were derived (Carterson et al., 2005).

The majority of studies have focused primarily on the microscopic analysis of conidial internalization and cell penetration of infected cells (DeHart et al., 1997; Paris et al., 1997; Wasylnka and Moore, 2002, 2003; Botterel et al., 2008; Han et al., 2011), the subsequent release of cytokines (Tomee et al., 1997; Borger et al., 1999; Kauffman et al., 2000; Zhang et al., 2005; Bellanger et al., 2009; Sun et al., 2012) and the activation of signaling proteins and pathways (Han et al., 2011; Balloy et al., 2008; Sharon et al., 2011). Transcriptome and proteomic analyses have recently been used to identify novel responses and pathways activated in infected cells (Gomez et al., 2010; Oosthuizen et al., 2011; Sharon et al., 2011; Fekkar et al., 2012). A major advance towards a more credible *in vitro* model has been the development of a cellular bilayer system constructed with human alveolar epithelial cells and human pulmonary artery endothelial cells grown on either side of a semipermeable polyester membrane (Hope et al., 2007; Gregson et al., 2012). There have been relatively few *in-vivo* studies analyzing the interaction between *A. fumigatus* and lung epithelial cells possibly because of the complexity of the immune response and the difficulty in getting reliable readouts from the epithelial cells alone (Kheradmand et al., 2002; Porter et al., 2009; Cunha et al., 2010; de Luca et al., 2010).

## The interaction of *A. fumigatus* with bronchial epithelial and nasal epithelial cells

**Figure 3:**
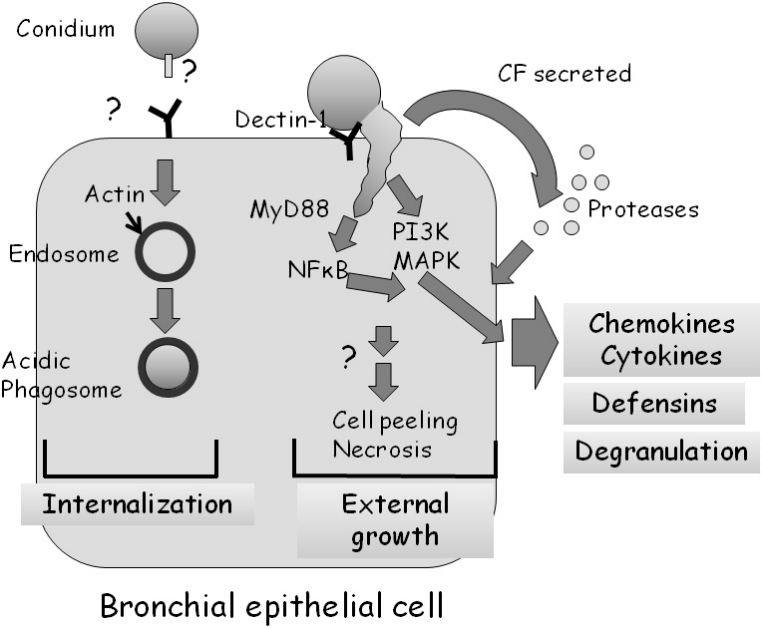
**Interaction of *A. fumigatus* with a bronchial epithelial cell.** Approximately 30% of adherent *A. fumigatus* conidia undergo internalization into endosomes which subsequently fuse to form acidic phagosomes. This process is dependent on the polymerization of actin around the endosome. Most ingested cells remain alive in the acidic phagosomes for up to 20 h. Approximately 70% of adherent *A. fumigatus* conidia germinate externally, activating MyD-dependent NFkB, PI3 kinase, and MAP kinase signaling, leading to chemokine and cytokine synthesis in a dectin-1 dependent manner. *A. fumigatus* infection also stimulates, through as yet unknown signaling pathways, the production of antimicrobial defensins and cell degranulation. Proteases secreted by the fungus also activate the production of cytokines.

Several groups have undertaken microscopic examinations of bronchial or nasal epithelial cells in culture, infected with live *A. fumigatus* conidia (Amitani et al., 1995; Paris et al., 1997; Botterel et al., 2008; Amitani and Kawanami, 2009). They demonstrated that approximately 20–50% of adherent conidia are internalized into late phagosomes where they remain ungerminated (Figure 3) (Botterel et al., 2008; Gomez et al., 2010). However, as most internalized conidia remain viable for up to 20 h, they may serve as a possible reservoir of infection. Externally adherent conidia proceed to germinate, causing damage to the cells (Figure 3). When grown on a biopsy of human bronchial tissue, hyphae penetrate through both intercellular and intracellular spaces of the epithelium, leading to loss of cilia and cell detachment (Amitani and Kawanami, 2009).

Balloy et al. (2008) examined the signaling pathways activated in bronchial epithelial (BEAS-2B) cells after conidial infection. Germinating but not resting conidia of *A. fumigatus* activated phosphatidylinositol3-kinase (PI3K), p38 mitogen activated protein kinase (MAPK), and ERK1/2 leading to interleukin (IL)-8 synthesis (Figure 3). The MyD88 pathway was also activated by *A. fumigatus*, leading to NF-κB activation; however, IL-8 production was not dependent on the TLR-MyD88 pathway. Therefore, two independent signaling pathways are activated in BEAS-2B cells by *A. fumigatus*, one that is MyD88/NF-κB-dependent and another that is PI3K/p38/ERK1/2-dependent and involved in IL-8 synthesis.

Human bronchial epithelial cells apparently recognize and respond to *A. fumigatus* through dectin-1 receptors that bind glucan exposed on the fungal surface (Cunha et al., 2010; Sun et al., 2012) by generating reactive oxygen species, antimicrobial peptides and cytokines (Figure 3). Inhibition of dectin-1 expression by RNAi blocked these responses. Interestingly, dectin-1 is present at low levels on the surface of these cells under normal conditions and is strongly upregulated in a TLR2-dependent manner in response to germinating conidia (Sun et al., 2012). This suggests that HBE cells have the ability to distinguish between dormant and germinating conidia and can increase the sensitivity and strength of their response over time.

The transcriptional response of bronchial epithelial cells in culture to infection by *A. fumigatus* conidia has been recently analyzed (Gomez et al., 2010; Oosthuizen et al., 2011). Gomez et al. (2010) analyzed the response of the specific subset of HBE cells that had internalized conidia following 6 h of infection. These cells had increased levels of transcripts from genes associated with repair and inflammatory processes (e.g., matrix metalloproteinases, chemokines, and glutathione S-transferase). Also enriched and upregulated were genes involved in chromatin assembly, G-protein-coupled receptor binding, chemokine activity, and glutathione metabolic processes, consistent with their established role in responding to fungal invasion in myeloid cells (Cortez et al., 2006). Genes involved in cell cycle phase, mitosis, and intracellular organelles were downregulated, suggesting that the infected cells reduced their rate of proliferation in response to direct interaction with conidia. Oosthuizen et al. (2011) analyzed the transcriptional response of both HBEs and *A. fumigatus* during infection. The response of the infected HBE cells was weak and did not overlap well with the results reported by Gomez et al. (2010). However, in both studies the innate immune response and the production of chemokines were increased.

Only a single publication has analyzed the proteomic response of *A. fumigatus*-infected bronchial (BEAS-2B) cells, limited to identifying the secreted proteins (Fekkar et al., 2012). Seven secreted proteins of human origin were significantly increased following infection, including three of lysosomal origin and four participating in the thioredoxin system. The release of lysosomal enzymes was dose-dependent and activated only by live conidia. It was partly dependent on the PI3K and p38-MAPK pathways. This result suggests that bronchial cells react to fungal infection by lysosomal degranulation and the release of proteins belonging to the redox detoxification system that may protect them from damage caused either by infection or by the host inflammatory response. However, at least in this cell-culture-based study, the fungus completely overwhelmed the cells and their degranulation did not seem to cause damage to the fungus.

Beta-defensins (hBDs) are antimicrobial peptides secreted by the lung epithelium in response to pathogens (Tecle et al., 2010). When bronchial or alveolar (A549) cells were infected with live swollen conidia they responded by secreting the defensins hBD2 and hBD9. However, the concentration of secreted defensins was too low to affect fungal growth in this model system.

In summary, the studies described here confirm that infected airway epithelial cells are not passive; they respond offensively by secreting lysosomal enzymes, antimicrobial peptides, and cytokines that alert and activate the immune system.

A methodological limitation of these experiments is that it is not possible to follow *A. fumigatus* cell infection for longer than ~24 h. After that period, profuse hyphal growth causes the cells to detach and die. Therefore, CF derived from mature mycelial culture has been used as an imperfect substitute to simulate some aspects of late infection. CF is prepared from *A. fumigatus* grown in cell culture medium for 2 days to a week. The mycelium is then removed, the medium is filtered to remove microorganisms and added to the epithelial cells in culture (Amitani et al., 1995; Tomee et al., 1997; Zhang et al., 2005; Sharon et al., 2011). When CF was added to primary ciliated nasal cells, it slowed ciliary beat frequency and caused epithelial disruption. Fractionation of the CF showed that the active fraction consisted of gliotoxin and other uncharacterized high-molecular-weight factors secreted by the fungus (Amitani et al., 1995). If these factors are produced in sufficient quantity *in vivo*, they could be important in the pathogenesis of *A. fumigatus* airway infections.

CF also induces the release of pro-inflammatory cytokines (IL-6, IL-8, and MCP-1) and mucin in treated H292 bronchial and primary nasal epithelial cells (Tomee et al., 1997; Kauffman et al., 2000; Oguma et al., 2011). Cytokine release, mucin secretion and cell peeling were blocked by addition of serine protease inhibitors, suggesting that secreted fungal proteases were responsible for these effects. By causing cell detachment, fungal proteases may decrease the ability of the epithelium to function as a physical barrier. In response, the epithelium may initiate an inflammatory mucosal response against *A. fumigatus* by releasing cytokines and secreting mucin.

The role of secreted fungal proteases in the development of asthma was also demonstrated in a mouse model (Kheradmand et al., 2002; Porter et al., 2009). A complete asthma phenotype, characterized by airway hyperresponsiveness, airway eosinophilia and recruitment of IL-4- and IFN- γ-secreting cells did not fully develop when mice were challenged intranasally with a protease-deficient strain of *Aspergillus niger* or allergen pretreated with protease inhibitors. These results suggest that as in cell culture, infected lung epithelial cells respond primarily to proteases secreted by the fungus, triggering an allergic response.

An important aspect of the lung response to pathogens, especially in allergy, is that the initial inflammatory response must be dampened, to enter a state of immunological tolerance. de Luca et al. (2010), recently demonstrated that addition of *A. fumigatus* conidia to primary bronchial cells activated the secretion of kinurenine, an anti-inflammatory molecule. Kinurenine is synthesized from tryptophan by the enzyme indoleamine 2,3 dioxygenase (IDO). IDO was shown to be activated in these cells by the TLR3-TRIF- NF-κB pathway, as bronchial cells from knockout mice in these genes, did not release kinurenine in response to *A. fumigatus* conidia. Alveolar inflammation and neutrophil influx in the Trif^−/−^ mice was strongly increased as compared to normal mice.

## The interaction of *A. fumigatus* with the alveolar epithelium

**Figure 4:**
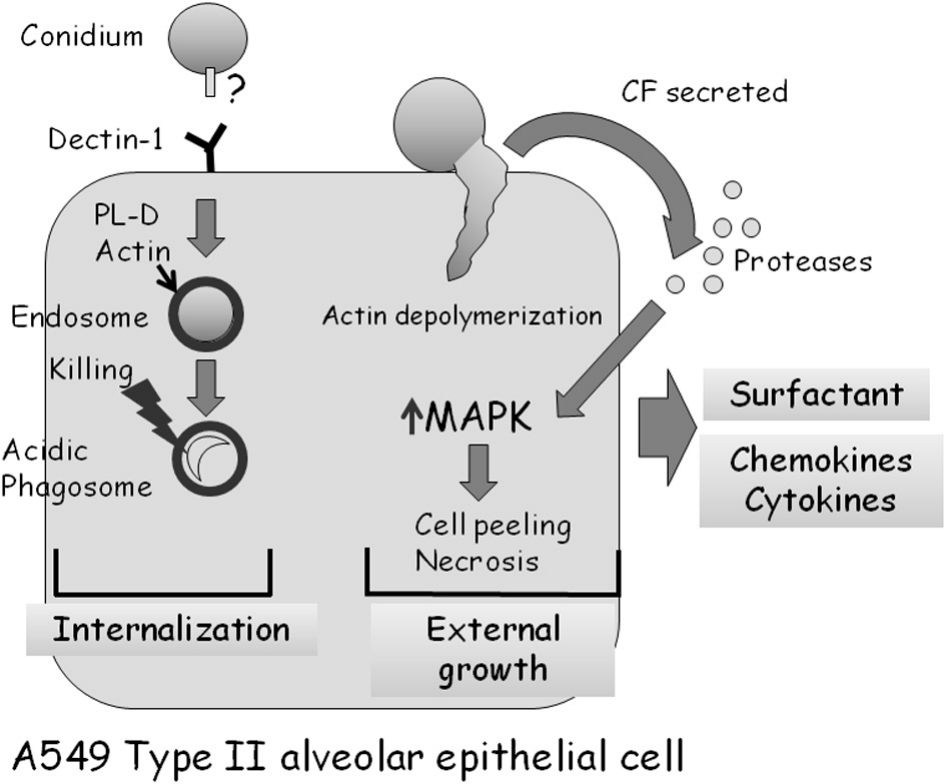
**Interaction of *A. fumigatus* with an A549 type II alveolar epithelial cell.** Approximately 30% of adherent *A. fumigatus* conidia are internalized into endosomes which subsequently fuse to form acidic phagosomes. This process is dependent on dectin 1 receptors on the cell surface, and the phospholipase-D dependent polymerization of actin around the endosome. Most (97%) ingested conidia are killed in the acidic phagosomes after 24 h. Approximately 70% of adherent *A. fumigatus* conidia germinate externally, causing actin cytoskeleton depolymerization and cell retraction. Proteases secreted by the fungus activate MAP-kinase signaling, leading to the production of cytokines and subsequently cell death by necrosis.

As described above, almost all studies analyzing the interaction of *A. fumigatus* with alveolar epithelial cells have been undertaken in cell culture using A549 cells, a cancer-derived (adenocarcinoma) cell line originating from type II cells (Bromley and Donaldson, 1996; DeHart et al., 1997; Paris et al., 1997; Tomee et al., 1997; Borger et al., 1999; Daly et al., 1999; Kauffman et al., 2000; Daly and Kavanagh, 2002; Wasylnka and Moore, 2002, 2003; Kogan et al., 2004; Zhang et al., 2005; Alekseeva et al., 2009; Bellanger et al., 2009; Han et al., 2011; Sharon et al., 2011). Early studies established that *A. fumigatus* conidia preferentially adhere to A549 monolayers and to the extracellular matrix they secrete (Bromley and Donaldson, 1996; DeHart et al., 1997). Infected cells form pseudopods surrounding some of the bound conidia followed by invagination and endocytosis (Paris et al., 1997). As in the bronchial airway epithelium, about 1/3 of adherent conidia are endocytosed, whereas the rest germinate externally, penetrating and damaging the cells (Wasylnka and Moore, 2002, 2003; Bellanger et al., 2009). During endocytosis, an actin ring encircles the endosome containing the conidium. Internalization is actin-dependent as it is blocked by depolymerization with cytochalasin D (Wasylnka and Moore, 2002). Conidia are trafficked into acidic phagosomes where only 3% survive after 24 h (Figure 4) (Wasylnka and Moore, 2003). Surprisingly, one third of these surviving conidia germinate and escape the phagosome, forming extracellular hyphae without lysing the host cell. Internalization seems to involve conidial binding to cell-surface dectin 1 receptors and activation of phospholipase D (PL-D) activity, which is inhibited by dectin-blocking antibodies or PL-D inhibitors (Figure 4) (Han et al., 2011). Taken together, these results suggest that A549 cells behave as non-professional phagocytes. They differ from macrophages and other professional phagocytic cells in being unable to efficiently process and destroy all ingested conidia.

The changes induced by conidial infection of A549 cells are not limited to the point of physical contact with the fungus. Live adherent conidia initiate rapid (<2 h) A549 cell retraction, loss of focal adhesions and the depolymerization of the entire F-actin cytoskeleton. This may enable the fungus to penetrate the thin underlying basal membrane layer more rapidly and enter the bloodstream (Kogan et al., 2004; Sharon et al., 2011).

Cytokine (IL-6, IL-8, TNF-α, GM-CSF, and MCP1) production is also stimulated in A549 cells in response to live conidia or mycelial fragments (Zhang et al., 2005; Bellanger et al., 2009; Sharon et al., 2011). However, unlike in bronchial cells, no studies have identified the signaling pathways involved. The central signaling kinases JNK and p38 are not phosphorylated in response to A549 cell conidial infection, whereas ERK1/2 phosphorylation is only weakly activated, suggesting that these kinases are not involved in responding to infection (Sharon et al., 2011). The whole-genome transcriptional response of infected cells indicates a general protective response, characterized by the activation of genes participating in intracellular signaling pathways and the secretion of inflammatory cytokines (Sharon et al., 2011).

The responses induced by *A. fumigatus* CF in treated A549 cells are very similar to those described for bronchial epithelial cells. The cells undergo a rapid loss of actin fibers and focal adhesions, followed by peeling and necrosis (Kogan et al., 2004; Sharon et al., 2011). Interestingly, these processes are blocked by addition of serine protease inhibitors or CF derived from protease-deficient *A. fumigatus* mutant strains (Figure 4). CF also induces the rapid phosphorylation and activation of the MAPKs ERK1/2, JNK, and p38. Activation depends on the protease activity of the CF, and does not occur in the presence of CF from a protease deficient strain of *A. fumigatus*. Strikingly, specific inhibition of ERK or JNK kinase activity strongly delays actin depolymerization, cell peeling and necrosis (Sharon et al., 2011). Together, these results imply that secreted fungal proteases, through an unknown mechanism, activate MAPK signaling in CF-treated A549 cells, leading to actin depolymerization, cell peeling, and necrosis. In addition, these findings suggest that inhibition of secreted fungal proteases or of the MAPK-mediated host response can decrease cellular damage, a finding with possible clinical implications.

Researchers have recently developed a bilayer model of the human alveolus that provides novel insights into *A. fumigatus* infection and treatment (Hope et al., 2007; Gregson et al., 2012). A549 alveolar cells and primary heart endothelial cells are grown on the upper and lower surface of a porous membrane, respectively, thereby mimicking the alveolar layers. GFP-fluorescent *A. fumigatus* conidia are seeded on the alveolar (A549 cell) side and their growth monitored. After about 15 h, some of the conidia are endocytosed but most germinate on the alveolar surface traversing both cell layers and the porous membrane between them. As the fungal hyphae pierce the lower endothelial layer, they secrete galactomannan into the endothelial compartment that can be quantified as a proxy for fungal invasion. Addition of macrophages to the top (A549 cell) compartment or Amphotericin B to the lower (endothelial) compartment partially delayed fungal invasion, whereas co-administration resulted in complete suppression of fungal growth (Hope et al., 2007). The alveolar bilayer model has also recently demonstrated its usefulness in addressing questions regarding the pharmacokinetics and pharmacodynamics of Amphotericin B formulations, itraconazole, and voriconazole (Lestner et al., 2010; Al-Nakeeb et al., 2012; Jeans et al., 2012). With further refinements, such as addition of type I epithelial cells, basal membrane proteins and various immune cells, this model can be used to simulate and parse the molecular signaling events occuring between the various cell types that make up a functional alveolus.

## Lung epithelial stimulation confers protection against *A. fumigatus* infection

In an exciting demonstration of the untapped therapeutic potential of lung epithelial cells, Evans et al. (2008) showed that mice pretreated with aerosolized bacterial lysates were highly protected against a broad array of bacterial pathogens, and also, surprisingly, against *A. fumigatus.* Protection against inhaled conidia occurred in immunocompromised neutropenic mice through the activation of multiple host defense signaling pathways in lung epithelial cells (Evans et al., 2008). These results suggest that immunodeficient patients at high risk to develop IPA, might benefit from stimulation of lung epithelial cell innate immunity. Additional approaches based on lung epithelial stimulation that have demonstrated promise in protection against bacterial infection include the overexpression of defensins (Shu et al., 2006), upregulation of NF-κB signaling pathways (Sadikot et al., 2006) or the administration of cytokines such as IL-22 and IL-17 that activate lung epithelial cell defenses (Aujla et al., 2008). These approaches may also provide protection against fungal lung infections.

## Summary and comparison

In summary, both bronchial and alveolar epithelial cells respond to *A. fumigatus* infection not as a simple physical barrier but as an extension of the innate immune system. They actively phagocytose a proportion of adherent conidia, and inhibit their growth. They produce cytokines and chemokines that activate both innate and acquired immunity and secrete antimicrobial peptides and lysosomal enzymes that may inhibit fungal growth. The signaling pathways involved in these processes have begun to be clarified. However, considerable gaps in our knowledge remain. Surprisingly, even less is known regarding the interaction of lung epithelial cells and other inhaled fungal pathogens such as *Cryptococcus neoformans*, *Paracoccidioides brasiliensis*, and *Histoplasma capsulatum*. *C. neoformans* apparently uses the capsular polysachharide GXM and secreted phospholipase B to bind to A549 cells via the CD14 receptor, leading to rapid internalization (45% after 1 h), cytokine and chemokine production and overwhelming cell lysis after 18 h (Barbosa et al., 2006, 2007; Ganendren et al., 2006; Guillot et al., 2008). *P. brasiliensis* binding to A549 cells is mediated by at least two laminin binding receptors and involves major cytoskeletal rearrangements in both the yeast and host cell. Internalization leads to A549 cell death by apoptosis after 24–48 h of infection (Mendes-Giannini et al., 2008). *H. capsulatum* is endocytosed by tracheal epithelial cells but the mechanism of recognition and cellular response remain unknown (Eissenberg et al., 1997).

Perhaps more insight can be gained by comparing the findings described in this review to the “state of the art” in the study of the interaction of the specialized bacterial pathogen *Mycobacterium tuberculosis* and lung epithelial cells (Krishnan et al., 2010). *M. tuberculosis* invades lung epithelial cells prior to dissemination. Heparin binding haemagglutinin adhesin (HBHA) enables *M. tuberculosis* to bind to sulphated glycoconjugates on epithelial cells. The bacteria undergo endocytosis into endosomes but escape killing by inducing phagosomal maturation arrest (PMA). Following intracellular replication, *M. tuberculosis* secretes lytic proteins (ESAT-6 and CFP-10) that lyse the infected cells enabling rapid bacterial spreading to the underlying endothelium and to adjacent epithelial cells.

It is doubtful however, that *A. fumigatus*, a non-specialized saprophyte, has evolved similar highly specialized mycobacterial-like adhesins and lytic proteins.

## Open questions

The field of lung epithelial cell research and *A. fumigatus* infection is likely to be advanced significantly by addressing the following unresolved issues: (1) at the systems level, who is the primary initiator of the lung inflammatory response to *A. fumigatus* infection? Is it the epithelial cells or the immune cells? What is the relative contribution of each? What are the main differences between the responses of bronchial and alveolar epithelial cells? How do they drive either an allergic Th2 response in ABPA or a Th1 cell mediated response in IPA? How do they interact and communicate bidirectionally with the different lineages of innate and adaptive immune cells to drive either one of these responses? (2) At the cellular level, do lung epithelial cells respond in a graded fashion to different morphotypes of the fungus or to increasing inoculum? How is the response downregulated? Is the apical response of lung epithelial cells different from the basolateral response? For example do they secrete antimicrobial peptides apically, toward the pathogen, and chemotactic mediators basolaterally, towards the blood vessels? How are gap junctions involved in host defense and cell–cell communication along the epithelial surface? (Thompson and Patterson, 2011) At the subcellular level-are Toll-like receptors involved in the lung epithelial response to *A. fumigatus* infection? How do secreted fungal proteases induce lung epithelial responses? Do they function by cleaving PAR-2 protease activated receptors? Do lung epithelial cells use additional mechanisms to disrupt fungal growth (i.e., synthesis of lysosyme, lactoferrin, calprotectin, SLPI protease inhibitors, etc.), and to initiate an inflammatory response (i.e., synthesis of leukotrienes and calprotectin). By what receptors and signaling pathways are they activated? Do these molecules also serve as ligands that bind to and activate/inactivate epithelial and immune cells?

I believe that by addressing these important questions, one can be optimistic for the development of novel intervention strategies aimed at specifically modulating the response of the lung epithelium to enable us to harness its defensive and offensive capabilities.

### Conflict of interest statement

The author declares that the research was conducted in the absence of any commercial or financial relationships that could be construed as a potential conflict of interest.
